# How does navigation system behavior influence human behavior?

**DOI:** 10.1186/s41235-019-0156-5

**Published:** 2019-02-13

**Authors:** Annina Brügger, Kai-Florian Richter, Sara Irina Fabrikant

**Affiliations:** 1 Department of Geography, University of Zurich, Winterthurerstr.190, 8057 Zurich, Switzerland; 2 Department of Computing Science, Umeå University, 90 187 Umeå, Sweden; 3 Department of Geography, University of Zurich, Winterthurerstr.190, 8057 Zurich, Switzerland

**Keywords:** Attention, Automation, Ecological validity, Empirical user study, Human–computer interaction (HCI), Incidental learning, Location-based services (LBS), Spatial cognition

## Abstract

Navigation systems are ubiquitous tools to assist wayfinders of the mobile information society with various navigational tasks. Whenever such systems assist with self-localization and path planning, they reduce human effort for navigating. Automated navigation assistance benefits navigation performance, but research seems to show that it negatively affects attention to environment properties, spatial knowledge acquisition, and retention of spatial information. Very little is known about how to design navigation systems for pedestrian navigation that increase both navigation performance and spatial knowledge acquisition. To this end, we empirically tested participants (*N* = 64) using four different navigation system behaviors (between-subject design). Two cognitive processes with varying levels of automation, self-localization and allocation of attention, define navigation system behaviors: either the system automatically executes one of the processes (high level of automation), or the system leaves the decision of when and where to execute the process to the navigator (low level of automation). In two experimental phases, we applied a novel empirical framework for evaluating spatial knowledge acquisition in a real-world outdoor urban environment. First, participants followed a route assisted by a navigation system and, simultaneously, incidentally acquired spatial knowledge. Second, participants reversed the route using the spatial knowledge acquired during the assisted phase, this time without the aid of the navigation system. Results of the route-following phase did not reveal differences in navigation performance across groups using different navigation system behaviors. However, participants using systems with higher levels of automation seemed not to acquire enough spatial knowledge to reverse the route without navigation errors. Furthermore, employing novel methods to analyze mobile eye tracking data revealed distinct patterns of human gaze behavior over time and space. We thus can demonstrate how to increase spatial knowledge acquisition without harming navigation performance when using navigation systems, and how to influence human navigation behavior with varying navigation system behavior. Thus, we provide key findings for the design of intelligent automated navigation systems in real-world scenarios.

## Significance

Envision that you exit a bus on your way to your friend’s house, but you have no idea where your friend’s house is. Luckily, you have your friend’s address on your phone, which is equipped with a navigation system. You confidently follow the route suggested by your smart device. As you arrive at your friend’s house, you discover that your friend is not there and that the battery of your phone is empty. On top of all this, you realize that you have lost your keys somewhere along the way. Would you be able to recall your path to the bus stop to search for your lost keys?

Navigation systems assist us during navigation, but they also affect our navigation behavior. During assisted navigation, we may completely rely on the system and tend to focus either on it or on matters other than navigation. We thus do not attend to the environment surrounding us, which degrades our spatial knowledge acquisition. Such behavioral changes are mostly unintentional and not properly empirically investigated, particularly in real-world environments.

In this study, we examine how navigation system behavior (in terms of automating cognitive processes) changes our behavior in and attention to the environment. We will only be able to design intelligent systems with a deeper understanding of their effects on human navigation behavior. Perhaps then the task of finding the same way back might not be as difficult as it was for some participants in our study.

## Introduction

The cognitive process of “navigation” consists of two major components: locomotion and wayfinding. Locomotion refers to the actual bodily motion of a human moving in his or her nearby surroundings. Wayfinding is the planning process of finding a destination. For example, using landmarks for orientation and decision-making (Montello, 2005). Finding a destination is an essential human behavior (Montello, 2005) and requires knowledge about the sequence of environmental properties, turns, segments, and sights along the route (Downs & Stea, 1973; O’Keefe & Nadel, 1978). To find our way from one place to another in partly or fully unknown environments, we nowadays often use automated navigation systems. Navigation systems primarily aim to deliver easy-to-understand navigation instructions that support people in reaching a destination more quickly and help reduce cognitive load during wayfinding (Allen, 1999). Despite the popularity of navigation systems, concerns have been raised in the literature about the negative effects on spatial knowledge acquisition caused by their extensive use (e.g., Gardony, Brunyé, Mahoney, & Taylor, 2013; Klippel, Hirtle, & Davies, 2010; Montello, 2005). These systems consume most of a pedestrian’s attention, leading to decreased spatial knowledge (Parush, Ahuvia, & Erev, 2007) and even to fatal accidents (Lin, Kuehl, Schöning, & Hecht, 2017) due to divided attention between the survey perspective offered by the navigation system and the route perspective, i.e., the first-person view (Gardony et al., 2013). As the trend towards using navigation systems increases, a considerable amount of literature has recently examined how navigation systems negatively affect spatial knowledge acquisition and human navigation behavior. For example, previous research (e.g., Münzer, Zimmer, Schwalm, Baus, & Aslan, 2006) comparing paper maps with navigation systems has found that pedestrians using paper maps show better spatial knowledge and orientation, but at the cost of lower navigation performance (e.g., longer duration to destination) compared to when they are using navigation systems. Despite considerable research demonstrating detrimental spatial knowledge acquisition with navigation systems (e.g., Bertel, Dressel, Kohlberg, & von Jan, 2017; Parush et al., 2007; Willis, Hölscher, Wilbertz, & Li, 2009), surprisingly few empirical investigations have been conducted about ways to balance navigation performance and spatial knowledge acquisition during assisted navigation. However, achieving such a balance seems feasible because navigation systems can feature varying levels of automation and, with this, vary the level of human involvement in decision-making. The influence of different navigation system behaviors—that is, different levels of automation—on human behavior is not yet understood. One of the main limitations of many empirical navigation studies is the missing connection to a real-world environment and, thus, their ecological validity (Dai, Thomas, & Taylor, 2018; Kiefer, Giannopoulos, & Raubal, 2013). This study empirically investigates the effect of different navigation system behaviors on human navigation and spatial knowledge acquisition in real-world navigation tasks in an urban, outdoor environment. One of the most important goals in designing different navigation system behaviors is to keep navigation performance high while increasing the user’s spatial knowledge acquisition. We will first briefly review the research investigating the impact of assisted navigation on spatial knowledge acquisition, which also motivates our research questions. We then introduce the empirical framework and study design. This is followed by a summary of the results of the study, which we critically discuss in the subsequent section. The paper ends with a summary of the implications of different automated navigation system behaviors on human behavior that should be considered when designing navigation systems and conducting outdoor studies.

## Background

### Spatial knowledge acquisition with navigation systems

Spatial knowledge acquisition has been discussed in several different research fields. Spatial knowledge was originally divided into three types: landmark, route, and survey knowledge (Siegel & White, 1975). However, critical arguments have emerged on whether the three types can really be (strictly) separated (e.g., Montello, 1998). Research has addressed how these types of spatial knowledge might change during navigation system use in different environments (e.g., Ishikawa, Fujiwara, Imai, & Okabe, 2008; Münzer et al., 2006; Parush et al., 2007; Willis et al., 2009).

For navigational tasks, acquiring spatial knowledge is crucial to orient and navigate in space without losing the way (Montello, 2005; Siegel & White, 1975). However, the formation of mental spatial representations is demanding and limited by humans’ attentional capacities (Downs & Stea, 1973; Münzer et al., 2006; Siegel & White, 1975; Wahn & König, 2017; Weisberg & Newcombe, 2018). Therefore, we often use a navigational aid to support the cognitive processes required to navigate as optimally as possible in an unknown environment (Ludwig, Müller, & Ohm, 2014). Several researchers have compared different kinds of navigation aids (e.g., Bakdash, Linkenauger, & Proffitt, 2008; Hirtle & Raubal, 2013; Ishikawa et al., 2008; Ishikawa & Takahashi, 2013; Klippel et al., 2010; Parush et al., 2007; Richter, Dara-Abrams, & Raubal, 2010; Willis et al., 2009). All found that modern digital navigation systems have a negative impact on the formation of mental spatial representations, but people using navigation systems are more time-efficient and effective in finding the route than people using paper maps (Dickmann, 2012; Lee & Cheng, 2008). On the one hand, a paper map can support tasks such as route planning, self-localization, and orientation (Thorndyke & Hayes-Roth, 1982), all of which require attending to the environment and acquiring information about environmental properties, such as spatial configuration and landmarks. On the other hand, navigation systems seem to change how humans attend to the environment, leading to a loss of the crucial skill of acquiring environmental knowledge (Parush et al., 2007). The use of a navigation system reduces what properties from the surroundings a navigator selects and diminishes the navigator’s allocation of attentional resources (Ishikawa et al., 2008). The navigation system designers pre-determine which properties get selected and how they are represented by the system, which, thus, also pre-determines the allocation of attention (Parasuraman, 2000).

During assisted navigation, the navigation system automatically selects and depicts environmental properties (e.g., landmarks) without any user intervention, which leads to decreased attentiveness to relevant properties (Taylor, Brunyé, & Taylor, 2008). Consequently, the navigator does not attend to the traversed surroundings, but reallocates attention toward the automated navigation system (Gardony et al., 2013; Willis et al., 2009). The resources are transferred toward the system itself and a respective instruction execution (Parasuraman, 2000). The navigator has to constantly switch between a survey perspective offered by the navigation system and a route perspective, i.e., the first-person view (Dai et al., 2018; Gardony et al., 2013). The distribution of human attentional resources changes with the use of navigation systems compared to no use of a navigation aid. Without a system, humans actively make decisions and interact predominately with their surroundings. Several studies have demonstrated that automated guidance divides a navigator’s attention between the navigation system and the environment (e.g., Gardony et al., 2013; Ishikawa et al., 2008). For example, a constantly updating GPS position signal (blinking light or beeping sound) on a navigation system can induce such an attentional division. As the navigator’s position is continuously updated, the visual tracking of the GPS signal distracts the navigator’s attention from the surroundings toward the system (Ishikawa et al., 2008). When continuously relying on this kind of positional updates, we do not attend to the information the traversed environment provides, and thus we lose the respective skill (Parush et al., 2007). But if the navigation system fails, navigators have to rely on their acquired knowledge, which would be challenging because not mentally processing properties along a travelled route results in decreased spatial knowledge in the end (e.g., Hirtle & Raubal, 2013; Huang, Schmidt, & Gartner, 2012; Münzer et al., 2006; Parasuraman, 2000; Parush et al., 2007).

To better understand attentional behavior in a spatial context, eye tracking is a technology that records a navigator’s gaze behavior during navigation (Duchowski, 2007; Holmqvist et al., 2011; Kiefer, Giannopoulos, Raubal, & Duchowski, 2017). Mobile eye tracking is particularly interesting in navigation scenarios because it can measure a human sense (gaze behavior) in real-world environments quite accurately and thus provides some indication of the information acquisition process (Kiefer et al., 2017). Fixation durations as an eye tracking measure can be interpreted as a measure of cognitive function and visual complexity of the scene (Duchowski, 2007; Goldberg & Kotval, 1999). However, the annotation process of the recorded data is laborious due to individual walking speeds and viewing directions in a constantly changing spatio-temporal context (Kiefer et al., 2017). Efficient methodologies to analyze such data are yet to be developed.

### Active role during spatial knowledge acquisition

An increasing number of empirical studies investigate how active and passive roles during navigation may influence the attention paid to the immediate surroundings of a navigator and, consequently, may support or hinder the formation of mental spatial representations. Münzer et al. (2006) introduced the active learning hypothesis. These authors contend that added active efforts during assisted navigation lead to spatial learning benefits. Attentiveness toward the environment (Klippel et al., 2010) and the level of control and the amount of decision-making (Bakdash et al., 2008) are suggested to yield differences in spatial knowledge acquisition. When actively making decisions and facing consequences, humans connect to their surroundings (Bakdash et al., 2008). Gardony et al. (2013) explored the relationship between navigators’ attention to the surroundings and their spatial decision-making during navigation system use. They discovered that if both decision-making with and attention to the traversed environment decrease, the navigators’ spatial knowledge acquisition also decreases.

Participants who knew that they had to learn a route (intentional learning) showed better route knowledge than participants who did not know that they would be asked to memorize a route (incidental learning; Chrastil & Warren, 2012). The ability of recalling objects for the two different learning types is different. Intentional learners are better at recalling the location of objects, and incidental learners are better at recalling the names of objects (Chrastil & Warren, 2012; Van Asselen, Fritschy, & Postma, 2006). Chrastil and Warren (2012, p.14) state that “(…) full route knowledge and survey knowledge appears to require the intention to learn, implying the need for attention to the relevant spatial relation. (…) intentional encoding appears to be necessary for place-action association, reproducing a route, and spatial relations between landmarks.” A navigation system directing attention to specific properties in the surroundings can lead to active encoding of spatial knowledge (Chrastil & Warren, 2012) and should be considered when designing such a system.

### Designing navigation systems that actively involve the user to increase spatial knowledge acquisition

During navigation system use, the locomotion component of navigation is emphasized over wayfinding, the planning and decision-making component (Montello, 2005)—planning and decision-making are essentially taken over by the system. A few scholars have begun to explore possible interventions during assisted wayfinding, such that users proactively make decisions to return their attention to the surroundings (Chung, Pagnini, & Langer, 2016; Kiefer, Giannopoulos, Sch, & Raubal, 2016; Parush et al., 2007). Such system interventions should be context-dependent, adaptable, and controllable by the navigator (Kiefer et al., 2016; Parasuraman, 2000; Richter, Tomko, & Cöltekin, 2015; Sheridan, 2002). However, Pielot and Rello (2017) found that system notifications distract a user and interrupt other activities. Moreover, Lee et al. (2014) found that smart devices featuring notifications increase the user’s attention allocation on the system and away from the surroundings. People seem to worry that they may miss important information if they are not attending to these notifications (Pielot & Rello, 2017). In contrast, a navigation system should invite a navigator to proactively attend to the environment (Kraft & Hurtienne, 2017) and thereby increase a navigator’s cognitive resource allocation for a task (Parasuraman, Sheridan, & Wickens, 2000), which in turn should lead to better spatial knowledge (Parush et al., 2007). To increase spatial knowledge acquisition, navigators need to interact with both their surroundings and the navigation system (Willis et al., 2009). Research suggests different cognitive problems using navigation systems and proposes several application solutions (Table 1).

**Table 1:** Two cognitive problems and their suggested solution based on Willis et al. (2009)

Cognitive problem	Solution
The passive nature of interaction	“Enable users to stimulate and control the delivery of information, and require users to confirm information mid-task” (p.109)
Lack of referencing between information delivered by application and real environment	“require user to either self-select cues from existing knowledge, self-report location or to cue-match so that the user cross-references features in the application with real-world environmental features” (p.109)

**Fig. 1:**
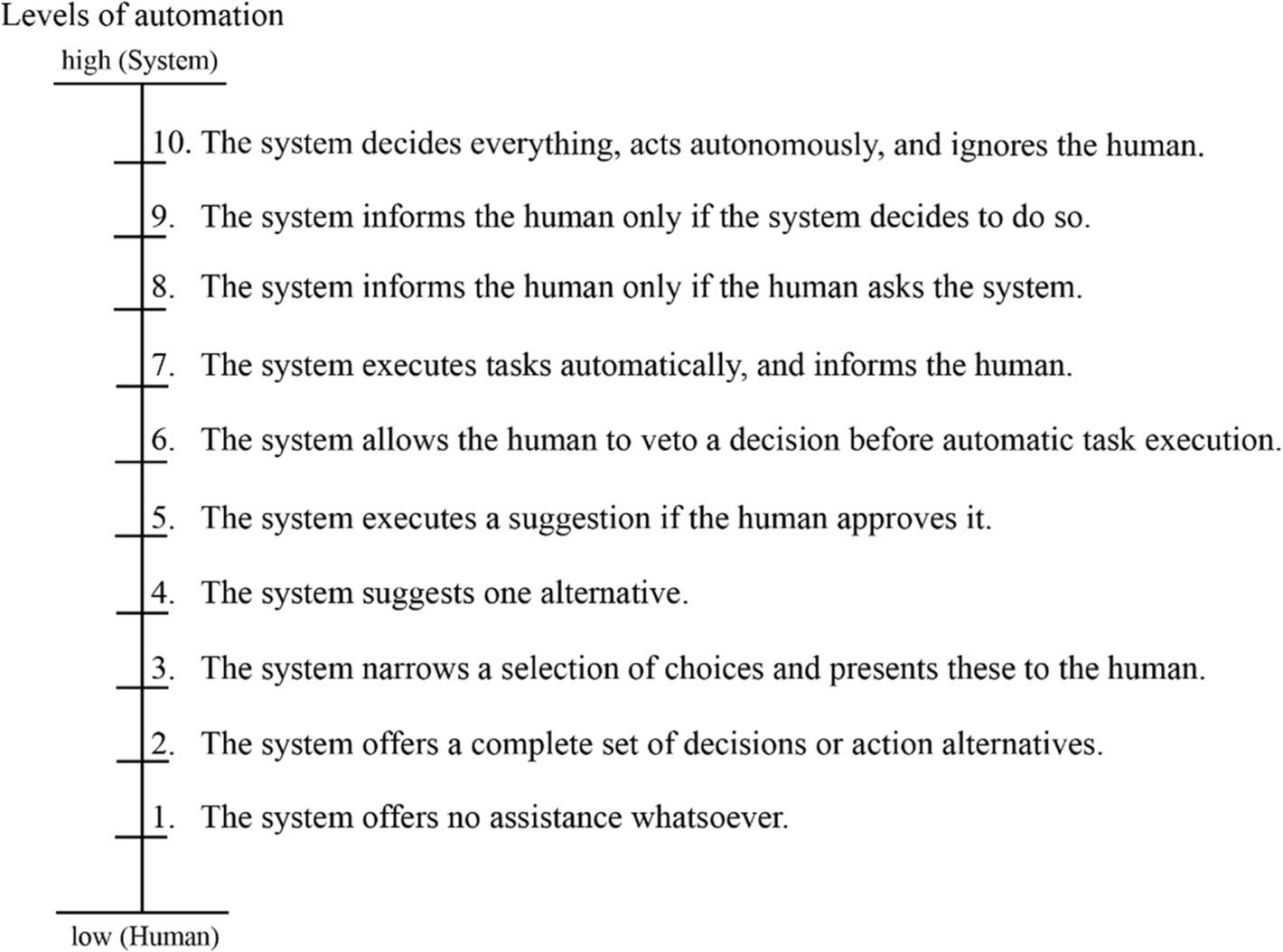
Levels of automation of decision and action selection (original figure in Parasuraman et al., 2000; adjusted text and extended with a linear scale): either the system decides and performs actions (high level of automation = System), or the human decides and performs actions (low level of automation = Human)

One way to do this is to engage navigators in a spatial location quiz, thus associating locations with a particular question (Parush et al., 2007), or to make them perform an otherwise automated action manually (Chrastil & Warren, 2012; Parasuraman et al., 2000) to improve mental representations. Parasuraman et al. (2000) identified different types (e.g., decisions or actions) and levels (from manual to full automation) of human interactions with automation. Figure 1 lists ten levels of automation for the decision and action selection. These levels vary from low (no system assistance; human makes all decisions and performs the actions) to high (system makes all the decisions; no human intervention possible).

All these studies clearly indicate that there is a relationship between system behavior and human behavior, and thus also with knowledge acquisition. However, it is unclear which kind of information and in what format a navigator ideally might need to get from a navigation system (e.g., Montello, 2009; Willis et al., 2009). Although some research has been carried out on spatial knowledge acquisition during navigation system use, very little has been done on how these findings translate to designing systems that assist navigators in both the navigational task and spatial knowledge acquisition, and, more generally, how pedestrians should engage with a system during navigation in outdoor environments (e.g., Dai et al., 2018; Giudice, Walton, & Worboys, 2010). The overall goal, according to Sheridan (2002), should be to design systems that complement humans.

The study presented in this paper empirically investigates human behavior during navigation tasks when facing different system behaviors according to the levels of automation as introduced by Parasuraman et al. (2000), using a novel empirical framework for efficiently testing pedestrians’ spatial knowledge in real-world environments (Brügger, Richter, & Fabrikant, 2016).

This study aims to answer the following research questions:

*Behavioral*: How do varying navigation system behaviors (levels of automation) influence (i) navigation performance, (ii) spatial knowledge acquisition, and (iii) gaze behavior during navigation tasks in a real-world outdoor environment? We hypothesize that when more automation is built into a navigation system, (i) the better the navigation performance, (ii) the lesser the spatial knowledge acquisition, and (iii) a change in gaze behavior occurs during navigation.

*Methodological:* Is the experimental framework of an assisted and unassisted navigation phase a valid approach to gather useful data in terms of spatial knowledge acquisition, and to allow for a smooth execution of an outdoor experiment? We hypothesize that the experimental framework of an assisted and unassisted navigation phase offers an efficient, ecologically valid way of determining spatial knowledge acquisition without the need for standard questionnaires and tests.

## Methods

This study aims to gain further insights into how navigation system behaviors influence human navigation and spatial knowledge acquisition. We conducted an empirical user study in an outdoor urban environment. We contend that the additional challenges of running studies in the real world are outweighed by the high ecological validity these settings offer (e.g., Kiefer et al., 2013). We applied a between-subject design by varying the behavior of the navigation system (independent variable) during an assisted route-following task. The dependent variables are (i) navigation performance, (ii) the acquired spatial knowledge during a route-reversal task, and (iii) gaze behavior.

### Participants

In total, 64 participants (44 females and 20 males), mostly freshmen at the University of Zurich and the ETH Zurich with different disciplinary backgrounds, took part in the experiment. The mean age of participants was 25 years, ranging from 18 to 60 years (M = 25 years, SD = 8 years). All except two participants owned a smartphone, thus representing a sample with background knowledge in using mobile digital devices. Each participant received CHF 20.00 for participation in the experiment. Participants signed a consent form approved by the Department of Geography at the University of Zurich and were told that they could stop the experiment at any time.

### Materials

**Fig. 2:**
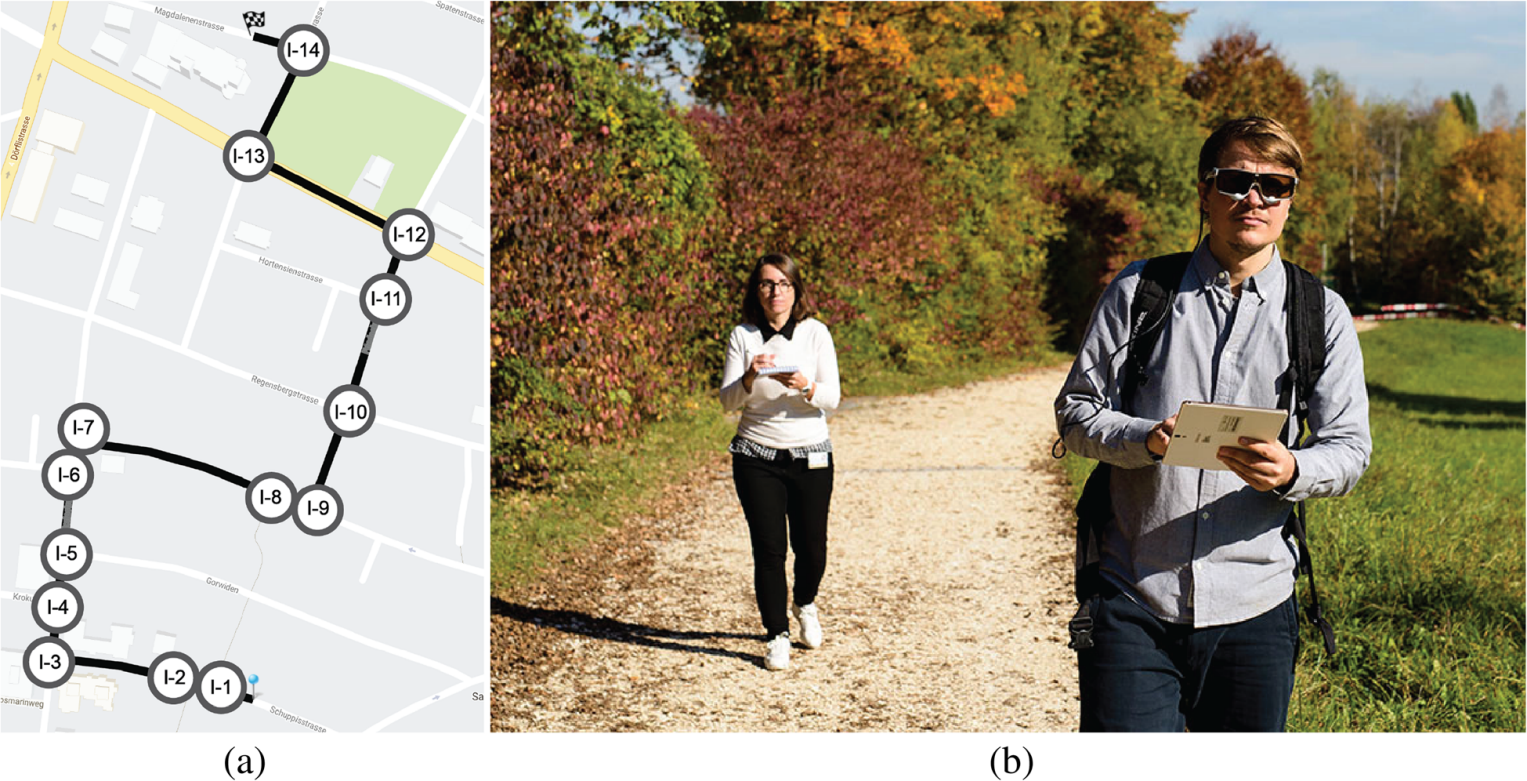
(**a**) Route (highlighted in *black*) with starting point (*blue pin*) and destination (*black flag*) depicted on the background map of Google Maps (© 2016 Google). All intersections are annotated with “*I-*” and a number indicating their position along the route. The intersection annotationss did not appear on the map display for the participants and are only added to this figure to allow references to intersections within this paper. (**b**) A participant holds the navigation system and wears eye-tracking glasses with an attached laptop in the backpack during the experiment. The experimenter follows the participant and takes notes (re-enacted scene). (Photo: Marc Latzel)

The study was conducted outdoors in Zurich, Switzerland. The study area is located in an urban residential neighborhood close to the University of Zurich, but it was unknown to the participants (we asked about familiarity in a questionnaire). The location of the route is displayed in Fig. 2a. The route was chosen by two of the authors based on its variety of intersections, turns, and landmarks. Figure 2a shows a Google Maps excerpt with the route highlighted in black. The blue pin at the bottom of the map indicates the starting point, and the black flag at the top of the map depicts the destination. The route is approximately 800 m long with a decline of 11 m in total. The route consists of 14 intersections (marked in Fig. 2a with “I-” and a number indicating its position in the route-following task) and different kinds of landmarks, building types, parks, etc., representing a typical urban residential environment. The route consists of three right (I-3, I-7, I-13) and three left (I-9, I-12, I-14) turns in walking direction from the start to the destination. The turns did not follow a regular pattern and divide the route in seven straight segments of different length.

A base map (Google Maps API) with the highlighted route was displayed on a SAMSUNG Galaxy Tab S10.2 tablet. The test application was set to display a north-up street map and did not allow for switching layers (e.g., to a satellite image) to ensure that all participants used the same road map. However, participants could rotate, zoom, pan, and tilt the map according to their needs to provide a map use experience very similar to that on their personal devices. In contrast to the original Google Maps available on mobile devices, the test map would remain in north-up orientation and at the initial zoom level if participants chose not to interact manually with it.

**Fig. 3:**
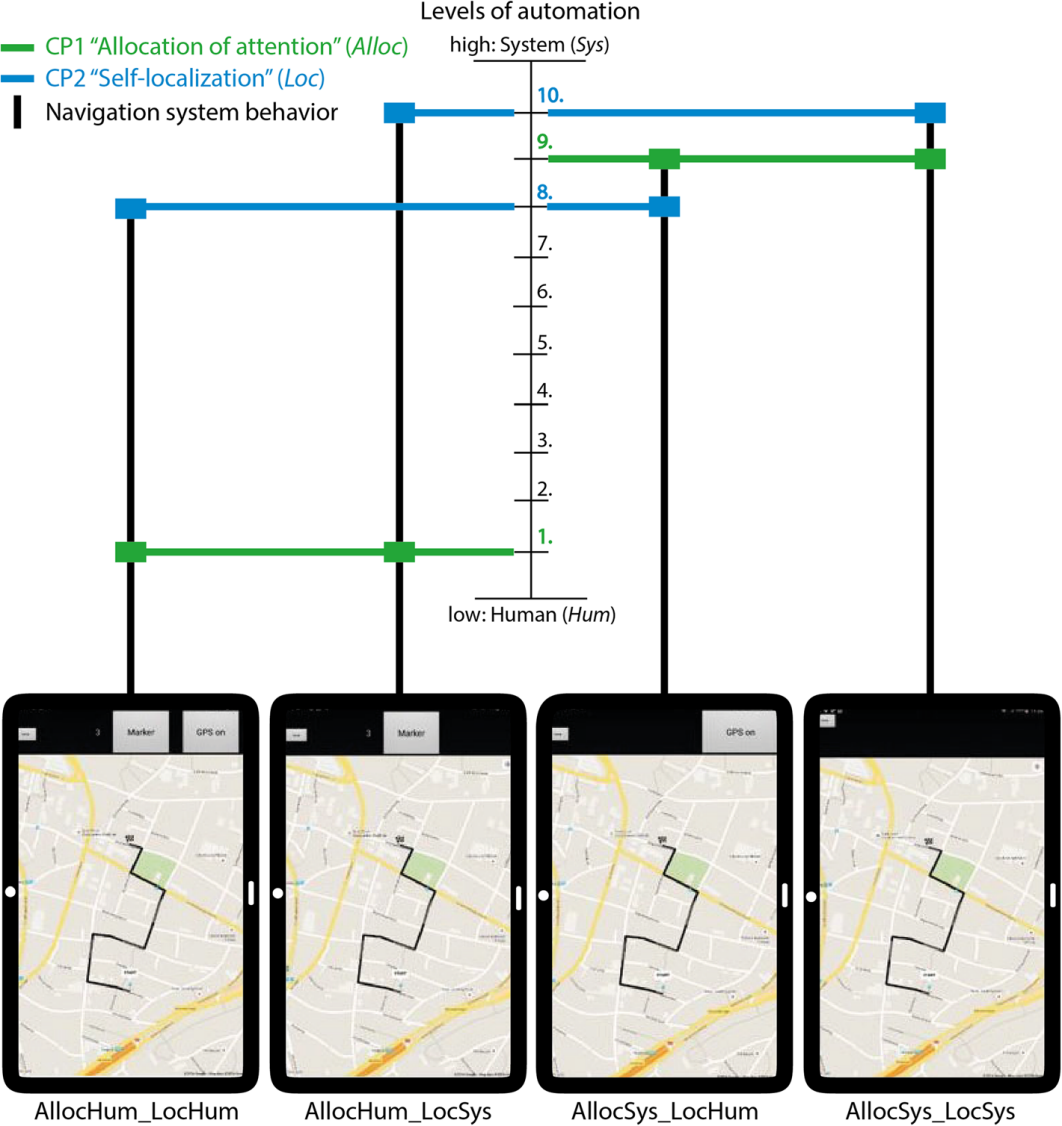
Four navigation system interface designs with varying navigation system behavior in terms of a combination of two cognitive processes (*CP*): allocation of attention (*Alloc*; *green*) and self-localization (*Loc*; *blue*). Each cognitive process involves either higher active participation from the human side (low level of automation; *Hum*) or higher system assistance (high level of automation; *Sys*). The figure illustrates for each navigation system design how the implemented processes map to the levels of automation of Parasuraman et al. (2000) (Fig. 1). The further up a horizontal bar is, the higher the level of automation. Background map is from Google Maps API (© 2016 Google)

We designed four navigation system variations that differ in their level of automation (i.e., system behavior). These variants combine two cognitive processes (CP), “allocation of attention” and “self-localization”, each in two different modes (detailed description below). Using these two modes is motivated by the reviewed research and by the role of attention during learning (e.g., Chrastil & Warren, 2012), the active learning hypothesis (Münzer et al., 2006), and the system solutions for cognitive problems (Willis et al., 2009) listed in Table 1. Combining the two cognitive processes, each with one of the two implemented modes, results in four different navigation system behaviors that we tested (Fig. 3). Each navigation system behavior is associated with either a high level of active participation on the human side and a low level of system automation (Fig. 3 left; “*Human*”, abbreviated “*Hum*”) or a high level of system assistance and a low level of active participation on the human side (Fig. 3 right; “*System*”, abbreviated “*Sy*s”). We used a between-subject design in which participants were randomly assigned to one of the four different navigation system behaviors, such that each system behavior was used by 16 participants.

*CP1: Allocation of attention* (abbreviated “*Alloc*”) directs one’s attention to a certain property in the environment, such as a landmark (Chrastil & Warren, 2012; Richter & Winter, 2014) and is implemented in the following modes to address the two cognitive problems stated by Willis et al. (2009) (Table 1).

- The system (abbreviated “*Sys*”) performs the process on its own, which means that the description of a certain landmark appears automatically on the map by displaying a marker symbol and a text description on the map as the user approaches it without any user interaction. The system vibrates for 5 s to make the user aware of the availability of this description and to make him or her attend to one of the three different landmarks used in this study (residential home, two adjacent flag poles, and a bus stop). This mode corresponds to “Level 9. The system informs the human only if the system decides to do so” by Parasuraman et al. (2000) as shown in Fig. 1.
- The system offers the opportunity for the human (abbreviated “*Hum*”) to type in some keywords that describe three self-chosen landmarks along the route. Participants are asked not only to make a decision regarding which landmark they wish to pay attention to, but also what kind of text they want to add at the chosen location. At three self-chosen locations along the route, participants were asked to press a “Marker” button in the app, which then allows them to type in a description of their current surroundings or some landmark in their vista space. The description is linked to the current position of the participant, but it does not appear on the map, i.e., the map does not change after performing this action. This mode corresponds to “Level 1. The system offers no assistance whatsoever” by Parasuraman et al. (2000).

*CP2: Self-localization* (abbreviated “*Loc*”) is the process of determining one’s current location in relationship to the environment by using visual clues (e.g., Meilinger, Hölscher, Büchner, & Brösamle, 2007). This was implemented in the following modes to address the two cognitive problems identified by Willis et al. (2009) (Table 1).

- The system (abbreviated “*Sys*”) performs the process on its own. This means that the location of the navigator is updated on the map as a blue dot and thus is permanently visible. This mode corresponds to “Level 10. The system decides everything, acts autonomously, ignores the human” of the levels of automation by Parasuraman et al. (2000) as shown in Fig. 1.
- The system provides the human (abbreviated “*Hum*”) with an opportunity to perform an action (pressing a “GPS on” button) to display the current location on the map for 10 s, after which the blue dot disappears. This mode corresponds to “Level 8. The system informs the human only if the human asks the system” of the levels of automation by Parasuraman et al. (2000).

Participants wore mobile SMI eye tracking glasses (SMI-ETG) during the experiment to record their gaze movement. Sunshades and wind protection stuck to the glasses reduced the infrared interferences and prevented participants from squinting. This protection was added to ensure better data quality from the eye movement recordings. The glasses were connected to a laptop that participants carried in a backpack. This laptop recorded all eye movements during the navigation experiment. Figure 2b shows the experimental setup, with a participant holding a navigation system and wearing a backpack with a recording device attached to the eye tracking glasses (for privacy reasons, a friend of the experimenter re-enacted the experimental scene).

### Experimental framework

We divided the experiment into two phases. During *Phase 1* (assisted route-following or “incidental knowledge acquisition”), we asked each participant to follow a route (Fig. 2a) presented by the navigation system (Fig. 3). Participants were first given a scenario that they had just left a bus at the starting point (blue pin in Fig. 2a) and that they had received a suggested route to a friend’s house (black flag in Fig. 2a). Participants were asked to follow this route as quickly as possible, without running. The two participant groups using the navigation system with a low level of automation in terms of the cognitive process “allocation of attention” were additionally given the following instruction (translated from German to English): “On the way to your friend’s house, you should write down three locations that are relevant for you for this route. The position at which this entry will be made is saved together with the text information and will be later integrated into the application. The entry is made when you click the ‘Marker’ button.” Importantly, all participants from all groups knew that there was going to be a second part of the experiment, but they did not know what this second task would involve. Therefore, the spatial knowledge acquired during the first phase can be considered the result of incidental learning.

For *Phase 2* (unassisted route-reversal or “knowledge recall”), we asked all participants to reverse the exact same route back to the starting point, similar in procedure to a study by Karimpur, Röser, and Hamburger (2016) that was conducted in a virtual reality (VR) setting. The scenario for this second phase was use-inspired. We told the participants that they had lost their keys, and because of a (fictitious) empty battery, they had to reverse the exact same route without using the navigation system. This also meant that the participants could not use any shortcuts in the route-reversal phase, even if they had been able to find them.

### Experimental procedure

The experimenter individually contacted participants and arranged a date for the experiment. The experiment took place during daytime from September to November 2016, on days without any rain. In case of forecasted rain, the experimenter cancelled and rescheduled the experiment because it was conducted entirely outside. Participants were asked to complete an online demographic questionnaire and the self-assessment questionnaire “Räumliche Strategie” by Münzer and Hölscher (2011) in advance at home. The questionnaire asked participants to rate their spatial strategies in global-geocentric, survey scale, and cardinal directions. Münzer and Hölscher (2011) showed that these self-report measures are able to predict participants’ spatial knowledge acquisition abilities. The experimenter sent a reminder to participants a day before the experiment and reminded them to fill out the questionnaire if they had not completed it by that date.

After arriving at the meeting point, participants were asked to sign a consent form and were introduced to the procedure of the experiment. Following this introduction, the experimenter explained the randomly assigned navigation system behavior (one of the four applications depicted in Fig. 3) to the participants, who then could get used to the application during a training session. Next, the experimenter asked participants to don the eye tracking glasses. This was followed by a three-point calibration phase with the eye tracking software iView. For the calibration of the eye tracking glasses, participants were asked to look at objects near them, such as street signs, in a distance of approximately 7–10 m. Participants who wear glasses were asked beforehand (via email) to wear contact lenses as a requirement for participation. The experimenter led the participant to the starting point of the test route where the participant was asked to read the instructions for Phase 1. The participant received the tablet with a running application, and was asked to perform the navigation task for Phase 1.

The experimenter shadowed participants at about 10 m distance, taking notes about potential changes in the environment for each participant. This was necessary because the experiment took place in a real dynamic urban environment (this point is taken up again in the Discussion section). After arriving at the destination, participants were given the instructions based on the scenario for Phase 2. Each participant was then asked to subjectively rate the difficulty of Phase 2 on a Likert scale from 1 (very easy) to 5 (very difficult). The rating before execution of this task provides a personal assessment of the perceived difficulty of the task, independent of a participant’s actual performance. Next, the participant was asked to reverse the route and walk unassisted (that is from memory) to the starting point of the route. Again, the experimenter shadowed participants at about 10 m distance. If participants took a wrong turn at an intersection (decision point), the experimenter had to call them back to the intersection where they had to make a new decision. Participants received explicit feedback (e.g., “You took the wrong road. Come back and make a new decision”) during their navigation performance (similar to Karimpur et al., 2016). After completing Phase 2, participants were asked 1) to draw the route on a printed map (the same map as shown on the starting screen, but without the route and starting and destination points), and 2) again rate the difficulty of Phase 2 on a five-point Likert scale. The participant rating indicates perceived navigation difficulty.

After completing the second phase of the experiment, participants were asked to fill out a post-test questionnaire and to complete the Building Memory test (Ekstrom, French, Harman, & Dermen, 1976). This test elicits an individual’s ability to memorize the position of buildings on a street map. Results of the test indicate a participants’ ability to memorize landmarks on a map (survey perspective) used during Phase 1, which in turn may explain parts of their performance of Phase 2. We administered the test after the main experiment so as to not give away the memory component of the experiment (Phase 2), which might have influenced their learning behavior during Phase 1. At the end of the experiment, participants received CHF 20.00 compensation, signed a confirmation of receipt, and were thanked for taking part in the experiment. The experimenter also reminded participants to keep the experimental procedure confidential. The experiment lasted about 70 min, on average.

### Mobile eye tracking analysis

**Fig. 4:**
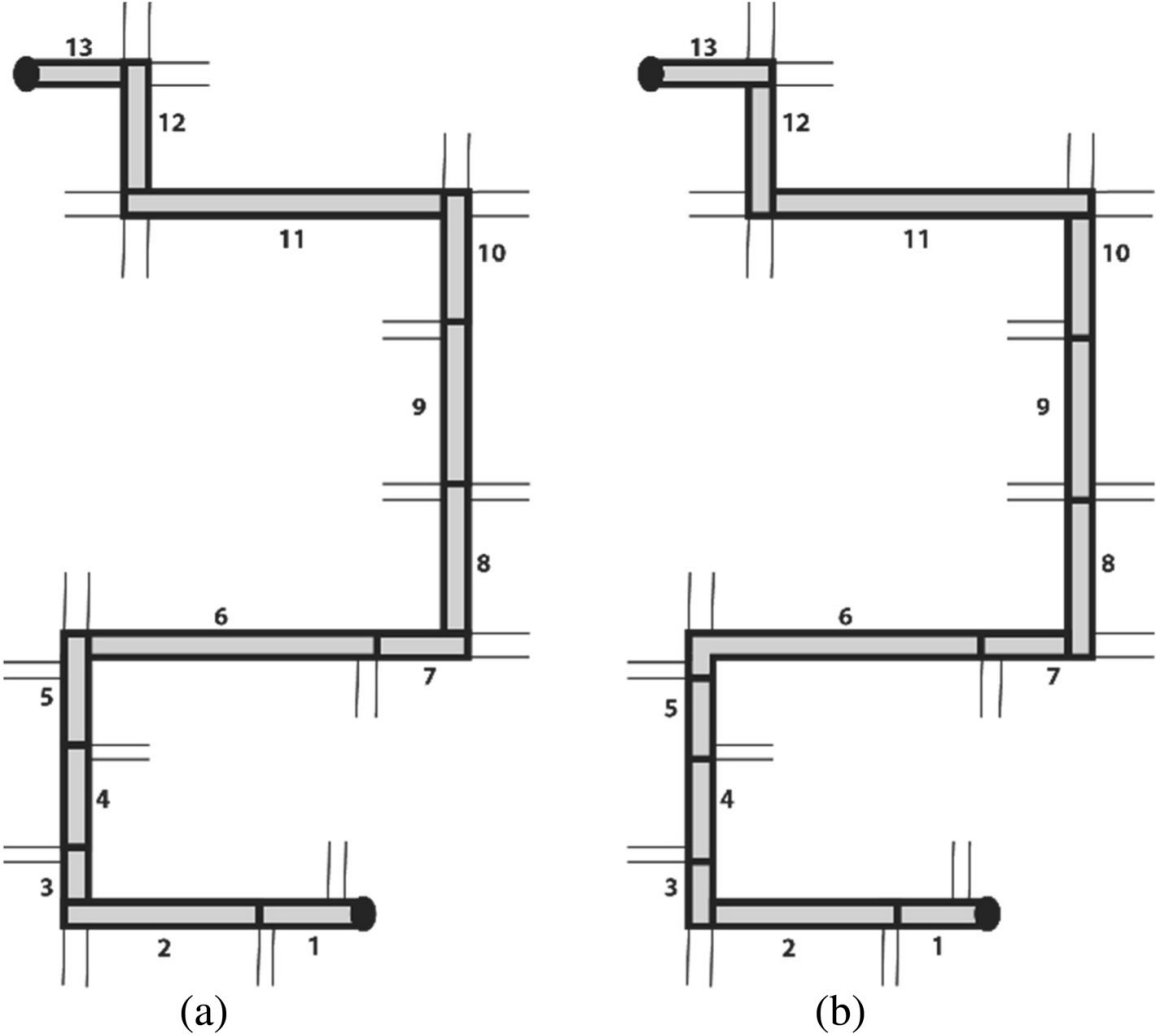
Spatial segmentation of the route (schematic) into 13 segments. Each segment represents the spatial context of approaching a decision point in walking direction including the relevant intersection. Spatial segmentation of the route for Phase 1 (**a**) and Phase 2 (**b**)

The first step in the eye tracking analysis was to segment the data such that it allows for comparison between participants’ behaviors along the route. This was necessary because eye tracking data in real-world environments are not synchronized with any other sensor data and also are not synchronized between participants; behavior is highly dynamic and the recordings do not (automatically) provide any spatial references to the environment they are recorded in (i.e., at the same point in time two different participants might be at two very different locations along the route). We segmented the route at intersections that correspond to decision points for both experimental phases, according to Fig. 4. Each segment features the spatial context of approaching a decision point and the intersection itself, resulting in 13 segments both ways. The data analysis was performed with the iMotions© software using a duration dispersion-based fixation algorithm (fixation duration > 100 ms). We annotated the screen recordings of participants’ eye movements with start and end position for each segment to assign each fixation (its duration) to a specific route segment.

Each fixation has a duration which we used to compute the mean fixation duration of each participant in each segment by using the following formula:

$$ \overline{x}=\frac{1}{N}\sum \limits_{i=1}^N{x}_i $$

where $\overline{\mathrm{x}}$ is the mean fixation duration in the segment, N the total number of fixations in a segment, and x_i_ the duration of fixation i in this segment.

## Results

We first describe the participant sample, including demographics, self-assessed spatial strategies, and spatial memory abilities. Second, we present the navigation performance for the two experimental phases separately. To evaluate participants’ navigation performance, we use four standard measures suggested by Dillemuth (2005), and Meilinger, Franz, and Bülthoff (2012): time to task completion, interactions with the map (e.g., zooming, etc.), navigation errors, and the number of stops along the route. This is followed by the results of the gaze analysis recorded with the mobile eye tracking glasses during Phase 1 and Phase 2. We report results according to the four groups of the between-subject design. All figures and tables follow the same order of system behaviors provided above (Fig. 3).

### Participants

The experimenter randomly assigned each participant to one of the four experimental groups. Each group consisted of 16 participants (11 females, 5 males). As mentioned, in an online questionnaire, we asked participants to report their frequency of using any kind of map application on their mobile system with a five-point Likert scale. Most participants (87%) use their smart device several times a month for navigational purposes. Apart from the use of digital map applications, we asked them to specify their experience in mapping-related fields, such as map reading and cartography (map production). The majority (80%) had experience in using map applications on a mobile device and in reading maps in general. The majority (60 to 70%) of the participants rate their experience with Geographic Information Systems (GIS) and with orienteering as little or none. These results suggest a relatively homogenous sample of participants in terms of map use in general and experience in using digital maps, specifically.

#### Spatial abilities

We collected participants’ self-rated spatial abilities with the “Räumliche Strategie” questionnaire by Münzer and Hölscher (2011). Table 2 reports the mean ratings of this test for each application type group. The *egocentric orientation* scale evaluates how well a person knows directions and routes. The *survey scale* summarizes how well a person can build a mental map, and the *cardinal direction scale* assesses awareness of cardinal directions. Question 12 (“I am good in remembering routes and finding my way back without problems”) directly matches the second experimental phase (“unassisted route-reversal”). The scores range from “1: I don’t agree” to “7: I strongly agree”. The higher the scores, the better participants assess their ability. Overall, the results reveal that all three scales show a large range within all four groups. There are no significant differences in ratings across the groups for any of the scales (egocentric, F(3,60) = 5.12, *p* = 0.525; survey, F(3,60) = 0.604, *p* = 0.615; cardinal, F(3,60) = 2.13, *p* = 0.106), tested with a one-way ANOVA. A Kruskal-Wallis test reveals that ratings for question 12 were also not significantly different between the groups (H(3) = 2.3191, *p* = 0.508). Hence, in terms of spatial abilities, these results indicate a homogenous sample of participants across and within the four system behavior groups.

**Table 2:** No difference in participants’ self-assessed spatial strategies scores across groups (means and standard deviations)

Strategic scale	AllocHum_LocHum		AllocHum_LocSys		AllocSys_LocHum		AllocSys_LocSys	
Total	M = 4.28	SD = 1.42	M = 4.43	SD = 1.08	M = 3.81	SD = 1.27	M = 4.02	SD = 0.86
Global-egocentric orientation	M = 4.22	SD = 1.49	M = 4.30	SD = 1.02	M = 3.76	SD = 1.13	M = 3.95	SD = 0.87
Survey	M = 4.44	SD = 1.36	M = 4.64	SD = 1.19	M = 4.05	SD = 1.50	M = 4.27	SD = 1.04
Cardinal direction	M = 4.03	SD = 1.47	M = 4.34	SD = 1.53	M = 3.18	SD = 1.43	M = 3.53	SD = 1.16
Question 12	M = 4.62	SD = 1.89	M = 5.12	SD = 1.31	M = 4.18	SD = 1.97	M = 5.06	SD = 1.34

Strategic scales: total (questions 1–19), global-egocentric orientation (questions 1–10), survey scale (questions 11–17), cardinal direction (questions 18–19) and the specific question 12: “I am good in remembering routes and finding my way back without problems”

#### Spatial memory

For the “Building Memory” test (Ekstrom et al., 1976), participants were asked to place buildings on an empty street map after studying the same layout with the buildings shown. Zero points were assigned if all buildings were placed at wrong locations. If all buildings were correctly positioned, the maximum achievable score was 24. Table 3 lists participants’ average scores. The higher the test score, the more buildings were correctly located. All groups show high mean scores with rather small standard deviations. The group *AllocSys_LocSys* (allocation of attention and self-localization by system) is the only group with an average mean score of less than 20 and the one with the largest standard deviation. Overall, spatial memory ability of our participants is high. A Kruskal-Wallis test revealed no significant differences between the four groups (H(3) = 0.9761, *p* = 0.807).

**Table 3:** No differences in spatial memory scores for the “Building Memory” test (by Ekstrom et al., 1976) across groups (means and standard deviation)

	AllocHum_LocHum		AllocHum_LocSys		AllocSys_LocHum		AllocSys_LocSys	
Score	M = 21.07	SD = 2.64	M = 20.61	SD = 3.17	M = 20.42	SD = 3.71	M = 18.98	SD = 5.02

The results of the spatial strategies and spatial memory tests indicate a homogenous distribution of spatial abilities across the four participant groups.

### Phase 1: Assisted route-following (incidental knowledge acquisition)

The first set of analyses examined the impact of different navigation system behaviors on human navigation behavior during Phase 1 of the experiment. This included navigation efficiency, stops and hesitations (i.e., significantly slowing down) along the route, and the interactions with the map during the route-following task. The findings of Phase 1 might then explain potential differences in incidentally acquired spatial knowledge that was tested in Phase 2.

#### Navigation performance

**Fig. 5:**
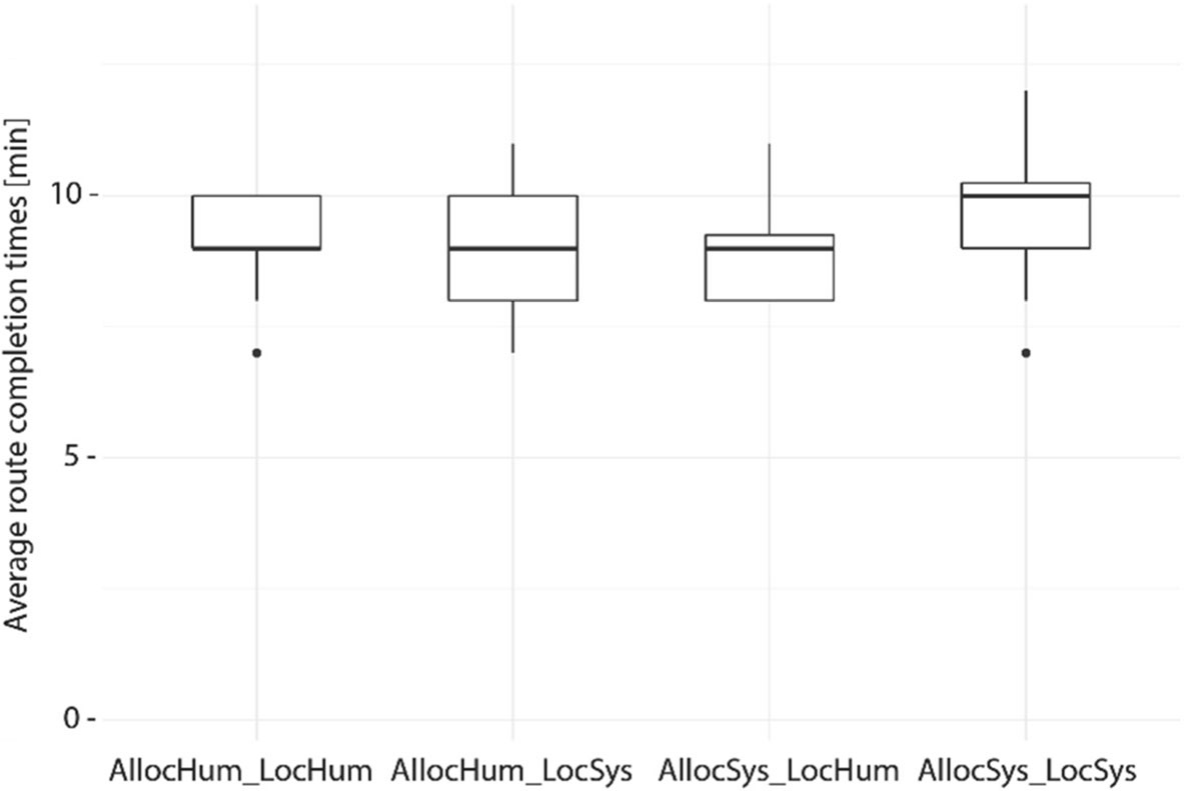
Navigation assistance levels do not influence route completion times for Phase 1. Average duration for walking the route assisted with a navigation system. *Black dots* indicate outliers

One goal of this study was to test whether a higher active participation of the human navigator with a navigation system (lower level of automation) could be achieved without harming navigation performance. Figure 5 depicts the duration for walking the route from the starting point to the destination assisted by a navigation system. Overall, the time to walk the route ranged from 7 to 12 min (M = 9.26 min, SD = 1.08 min). A Kruskal-Wallis test revealed no significant differences for completion time between the four groups (H (3) = 3.356, *p* = 0.339). This result shows that different system behaviors did not affect the time it took for participants to complete Phase 1. Furthermore, none of the participants made any navigation errors during Phase 1.

We analyzed how many times participants stopped or hesitated (slowed down) along the route during Phase 1. A stop means that the participant has both feet on the ground and does not move in any direction. A hesitation is a clearly identifiable reduction of speed while continuing to move. Overall, participants hardly ever hesitated during the assisted route-following phase. The two groups *AllocHum_LocHum* (allocation of attention and self-localization by human) and *AllocHum_LocSys* (allocation of attention by human and self-localization by system) stopped on average two to three times (to type the required keywords), but without harming their efficiency, as Fig. 5 shows.

**Fig. 6:**
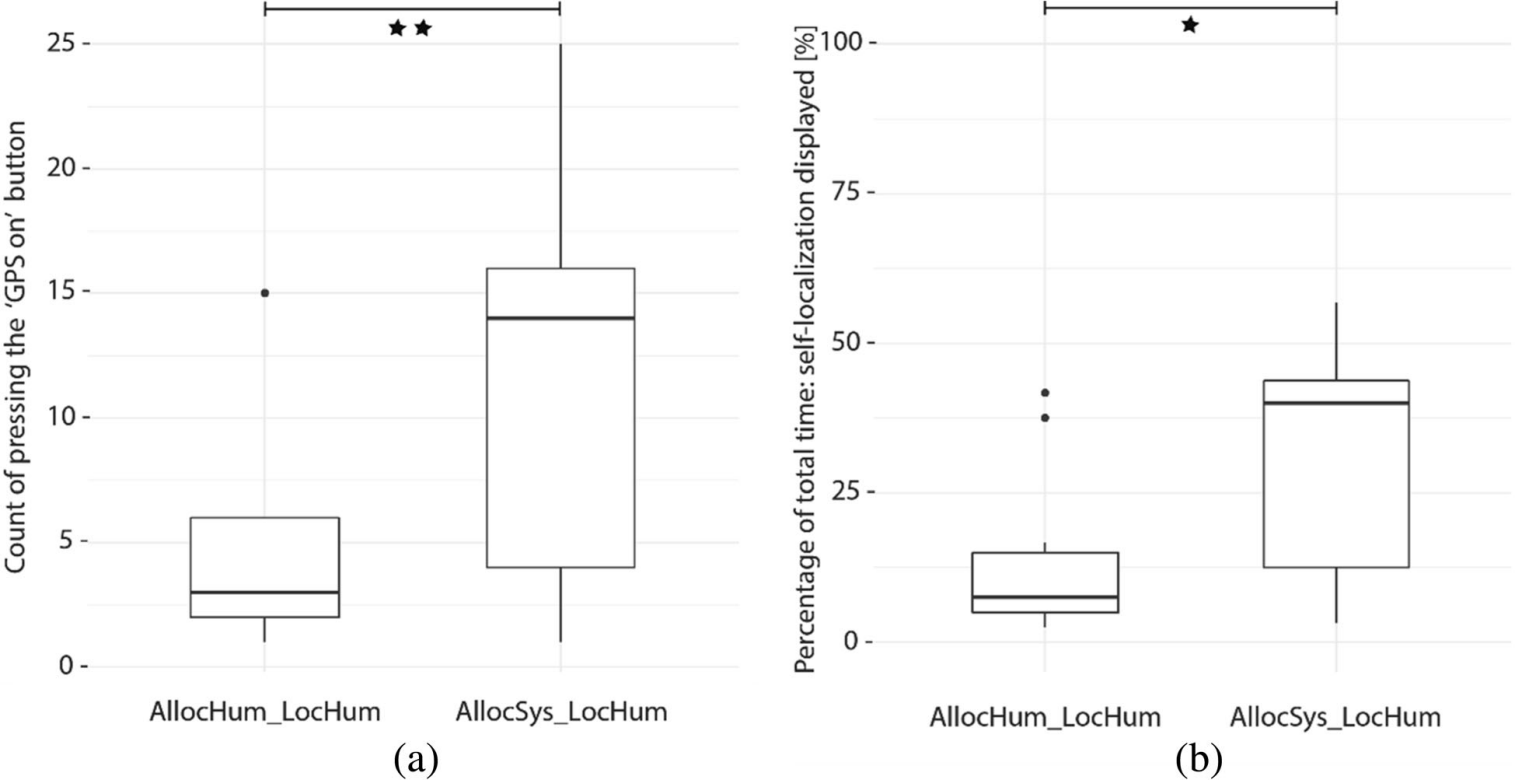
Participants in group *AllocSys_LocHum* pressed the “GPS on” button more often than the group *AllocHum_LocHum* (**a**)*.* Distribution within and across groups of counting the instances of participants pressing the “GPS on” button (statistically significant difference, **p < 0.01). Therefore, participants of the *AllocSys_LocHum* group had the self-localization displayed and accessible for a longer amount of time (statistically significant difference, *p < 0.05) (**b**). *Black dots* indicate outliers

As mentioned, two out of the four groups (AllocHum_LocSys, AllocSys_LocSys) were perpetually shown their position on the digital map while navigating. The other two groups (AllocHum_LocHum, AllocSys_LocHum; allocation of attention by system and self-localization by human) had the option to display their current location on the map by pressing the “GPS on” button. On the one hand, pressing this button causes a distraction from attending to a navigated surrounding if it is unnecessarily used. On the other hand, this can help to self-localize and reorient in the environment, if used strategically. Figure 6a suggests that, on average, the *AllocSys_LocHum* group (Mdn = 14, SD = 7.4) used the “GPS on” button more often than the *AllocHum_LocHum* group (Mdn = 3, SD = 4.9). This difference is statistically significant (W = 33.5, *p* < 0.01, r = − 0.51; Wilcoxon test). Therefore, also the time that the self-localization information was displayed was considerably higher for the *AllocSys_LocHum* group (Mdn = 40, SD = 18.2) than for the *AllocHum_LocHum* group (Mdn = 7.5, SD = 12.8) (Fig. 6). This difference is statistically significant (W = 31, *p* < 0.05, r = − 0.53; Mann-Whitney U test).

**Fig. 7:**
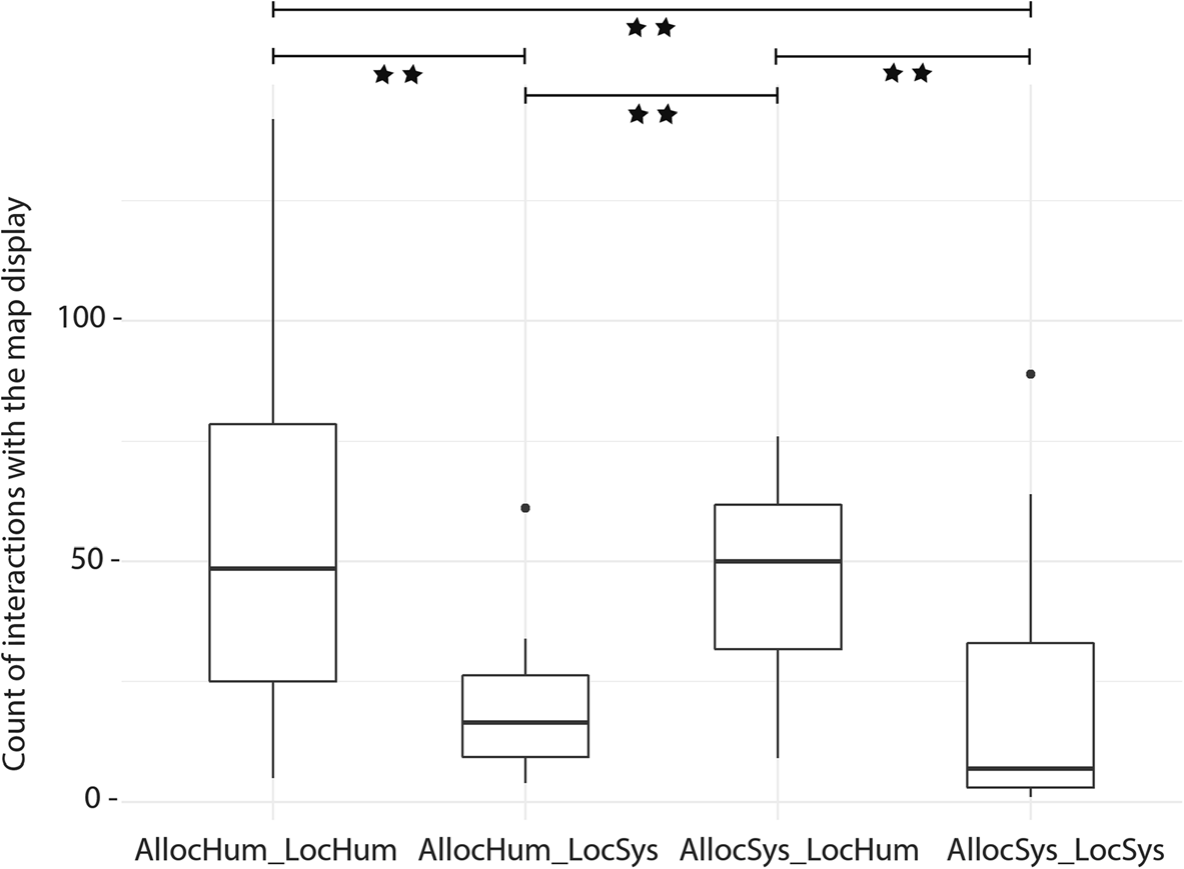
Significant differences in counts of interactions (zoom, pan, rotate) with the map display during Phase 1 across groups (statistically significant difference between groups, **p < 0.01). *Black dots* indicate outliers

Each time a participant zoomed, panned, rotated, or tilted the map, the system recorded the type of interaction in a log file. Figure 7 shows all the interactions with the navigation system, aggregated across the four navigation system groups. Generally, some participants interacted a great deal with the map display, while others hardly ever interacted with the map. None of the participants used the tilt function.

The group *AllocHum_LocHum* has the largest range of interactions. The group *AllocHum_LocSys* shows the smallest range, with one outlier (Fig. 7). A Kruskal-Wallis test suggests that the amount of interactions with the navigation system significantly differs between the four groups (H(3) = 18.166, *p* < 0.01). Pairwise comparisons of the mean ranks between groups reveal the following significant differences; the critical difference for all comparisons was 17.36 (corrected for multiple comparisons) at a 0.05 level (Table 4).

**Table 4:** Differences in interactions with the navigation system between groups

	AllocHum_LocHum	AllocHum_LocSys	AllocSys_LocHum	AllocSys_LocSys
AllocHum_LocHum	–	–	–	–
AllocHum_LocSys	19.75*	–	–	–
AllocSys_LocHum	1.34	18.40*	–	–
AllocSys_LocSys	21.15*	1.40	19.81*	–

Statistally significant difference between groups, *p < 0.05

### Phase 2: Unassisted route-reversal (knowledge recall)

First, we report the number of errors participants made in the route-reversal task, which reveals how well participants are able to recall their incidental spatial knowledge acquired during Phase 1. Second, we examine the efficiency of participants’ unassisted navigation by looking at duration of the route-reversal phase and counting of stops and hesitations along the route. Finally, we report participants’ self-reports of task difficulty collected in Phase 2, before and after completing Phase 2 to compare self-assessed task difficulty with actual task performance.

#### Testing spatial knowledge

We tested the participants on how well they found their way back to the starting point unassisted. Because participants were asked to reverse the exact same route to the starting point, each wrong turn at an intersection was counted as one error. Table 5 summarizes the results for the different groups. In the two groups with more active navigator participation *(AllocHum_LocHum* and *AllocHum_LocSys*)*,* three participants (18%) made a wrong route choice at one intersection. In the group *AllocSys_LocSys,* six (37.5%) participants made at least one mistake during Phase 2. What stands out is that 10 out of 16 participants (62.5%) in the group *AllocSys_LocHum* made a wrong navigation decision at at least one intersection. Table 5 also lists the number of errors per person and per group and the mean error per group. A Kruskal-Wallis test reveals that the mean error is significantly affected by the navigation system behavior (H(3) = 8.4962, *p* = 0.034). However, it is important to mention that the number of errors is often zero and generally low. Still, the number of participants with a navigation error varies greatly between the groups. The four participants with the highest number of errors (three) are in the two navigation groups using a navigation system that features lower active human participation (i.e., higher levels of automation). More errors suggest that these participants were less effective in recalling their spatial knowledge of the route compared to the other participants, and indeed acquired less (accurate) spatial knowledge during Phase 1. Twelve participants made only one error, and 42 participants made no errors at all. Hence, these participants were more effective in reversing the route.

**Table 5:**
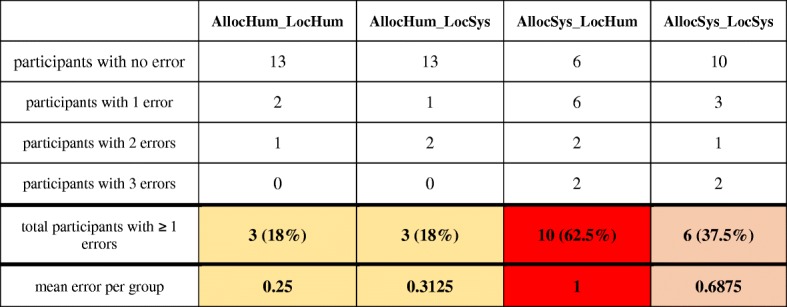
Different numbers of navigation errors across groups during the experimental Phase 2 indicating varying degrees of recalling the acquired spatial knowledge of the traversed route

Number of participants per group who made zero, one, two or three errors at intersections (darker red color indicates more navigation errors per group)

#### Navigation performance

**Fig. 8:**
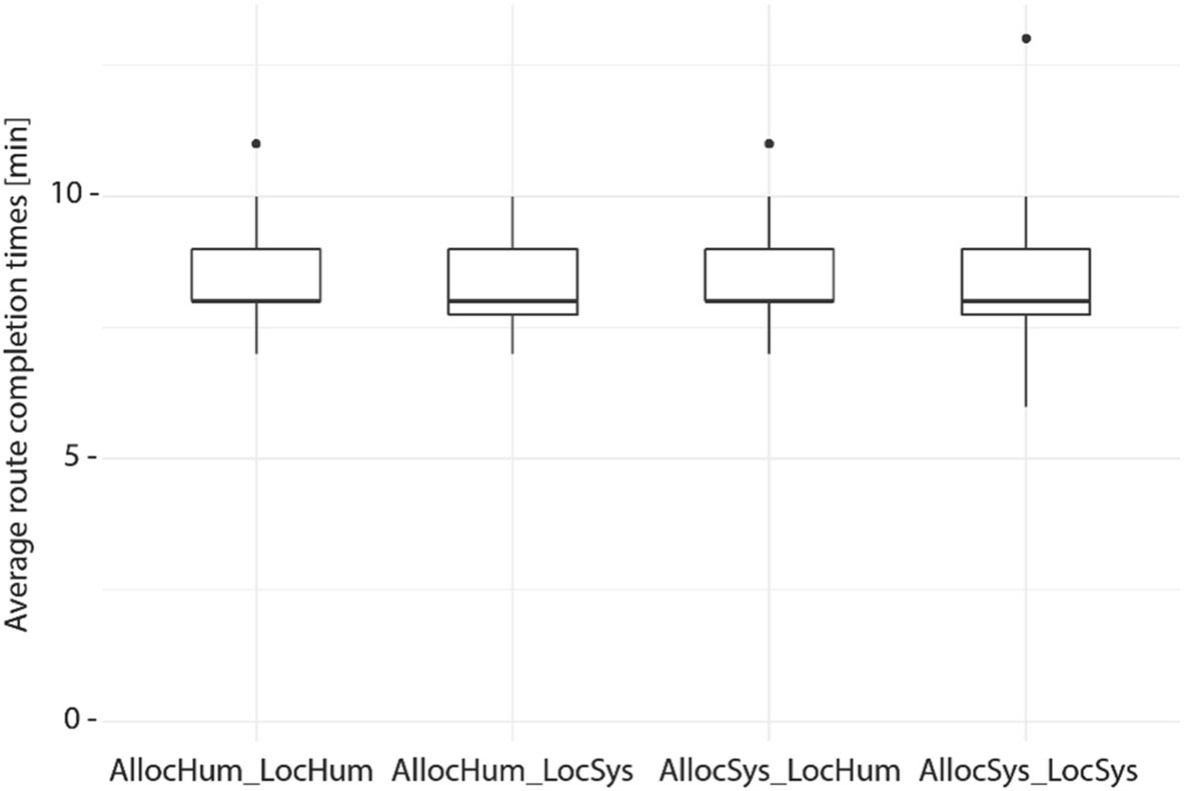
Navigation assistance levels do not influence route completion times of Phase 2. Average duration for walking the route unassisted. *Black dots* indicate outliers

Figure 8 depicts the duration for reversing the route unassisted from the destination back to the starting point. Overall, the time to walk the same route unassisted ranged from 6 to 13 min for participants (M = 8.3 min, SD = 1.2 min), with most participants returning to the starting point in less than 10 min. A Kruskal-Wallis test revealed no significant completion time differences between the four groups in Phase 2 (H(3) = 0.051, *p* = 0.997). This means that being exposed to differing navigation system behaviors during Phase 1 did not significantly influence navigation performance without any navigation system assistance for the reversed route (Phase 2).

Similar to Phase 1, we counted how many times participants stopped or hesitated along the route during Phase 2. On average, participants hesitated zero to once across groups (Table 6). The two groups *AllocSys_LocHum* and *AllocSys_LocSys* stopped slightly more often than the groups *AllocHum_LocHum* and *AllocHum_LocSys*, who hardly ever stopped or hesitated during unassisted navigation phase.

**Table 6:** Count of hesitations and stops across groups during the unassisted route-reversal task (mean and standard deviations)

	AllocHum_LocHum		AllocHum_LocSys		AllocSys_LocHum		AllocSys_LocSys	
Hesitations	M = 0.50	SD = 0.63	M = 0.43	SD = 0.51	M = 1.31	SD = 1.19	M = 1.06	SD = 1.61
Stops	M = 0.12	SD = 0.34	M = 0.18	SD = 0.40	M = 0.56	SD = 0.89	M = 0.37	SD = 0.61

Participants barely hesitated or stopped in Phase 2. The groups *AllocSys_LocHum* and *AllocSys_LocSys* hesitated slightly more often than the other two groups, indicating uncertainty in recalling the next navigation action or in recognizing the surrounding environment

#### Difficulty rating

After participants had read the instructions for Phase 2, they were asked to rate their perceived difficulty of the task “finding the exact same way back without assistance” on a five-point Likert scale ranging from 1 (very easy) to 5 (very difficult). They were asked to rate the difficulty of the task again after completing their walk back, using the same scale. Table 7 shows the average scores across the four groups. Overall, on average, the ratings are all below 3, thus indicating they perceived the task to be easy. The range of ratings is larger before than after participants performed the route-reversal. The variation in ratings is very small for group *AllocSys_LocSys*, meaning that participants in this group agreed more about the difficulty of this task before and after Phase 2 compared to the other groups. All groups rated the difficulty of Phase 2 as easier after they performed it compared to before. This indicates an overestimation of task difficulty in their first rating. A Kruskal-Wallis test revealed no significant differences in ratings before (H(3) = 3.6814, *p* = 0.289) or after (H(3) = 0.75636, *p* = 0.8599) performing Phase 2 across the four groups.

**Table 7:** Average score of task difficulty across groups at different stages (before and after) in Phase 2

Difficulty Rating	AllocHum_LocHum		AllocHum_LocSys		AllocSys_LocHum		AllocSys_LocSys	
Before Phase 2	M = 2.43	SD = 1.15	M = 2.37	SD = 1.14	M = 2.97	SD = 0.92	M = 2.81	SD = 0.75
After Phase 2	M = 2.12	SD = 0.8	M = 2.12	SD = 0.95	M = 2.43	SD = 0.15	M = 2.31	SD = 1.07

Rating scale: 1 (very easy) to 5 (very difficult). All groups rated the difficulty of Phase 2 as easier after they performed it compared to before, indicating an initial overestimation of task difficulty

### Mobile eye tracking

Overall, the differences in navigation performance and spatial knowledge acquisition during Phase 1 and Phase 2 indicate changes to human navigation behavior based on navigation system behavior. To see if navigation system behavior also influences gaze behavior, we now report the analysis and results of the eye tracking recordings during Phase 1 and Phase 2. Unfortunately, we could only analyze 26 of 64 participant recordings (*AllocHum_LocHum*, 6; *AllocHum_LocSys*, 6; *AllocSys_LocHum*, 8; *AllocSys_LocHum*, 6) that had adequate data quality for both experimental phases due to calibration and recording issues. Given the small sample size in each group, we did not run any statistical analyses on the eye tracking data.

**Fig. 9:**
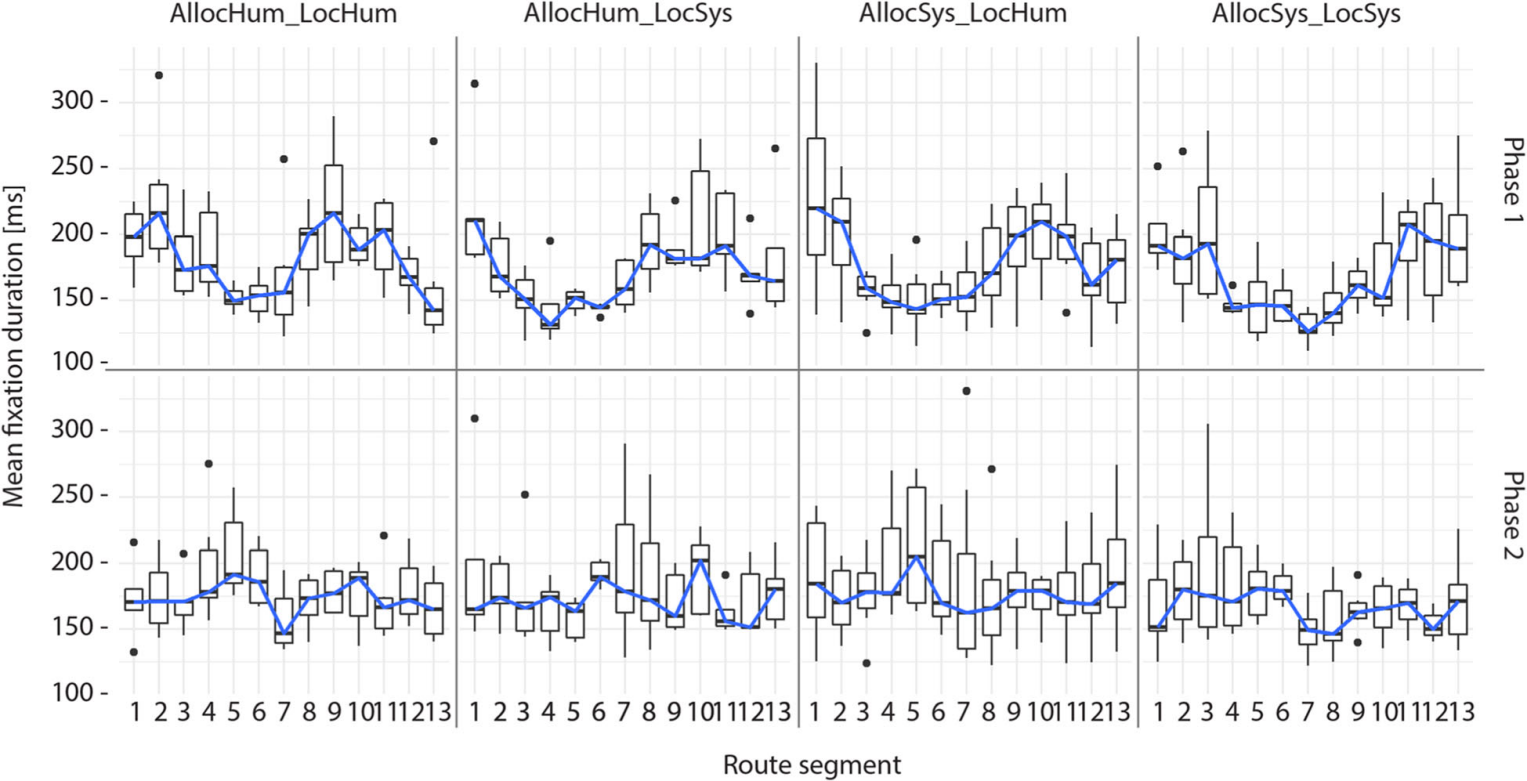
Mean fixation duration in each segment of the route across the four conditions for the experimental Phase 1 and Phase 2. For each participant the walking direction was from Segment 1 to Segment 13 in Phase 1 (i.e., read graph from left to right) and from Segment 13 to Segment 1 in Phase 2 (i.e., read graph from right to left). *Black dots* indicate outliers

Figure 9 shows the mean fixation durations for each segment during both test phases and across the four groups. What stands out first is that generally, for all groups, the mean fixation duration during Phase 1 (incidental knowledge acquisition) follows a wave pattern that starts with longer mean fixation durations in the first segments of the route, followed by segments with shorter mean fixation durations, and then again segments with longer fixation durations toward the end of the route. This wave pattern seems to be independent of the employed navigation system behavior. During Phase 2 (knowledge recall), we do not observe this wave pattern. Here, no clear pattern emerges, and fixation durations show large variations. Interestingly, the pattern of the *AllocSys_LocHum* group was inversed during Phase 2 compared to Phase 1.

However, while there do not seem to be differences between the four navigation system behaviors, distinct differences in the fixation duration patterns emerge between the two experimental phases: incidental knowledge acquisition (Phase 1) and knowledge recall (Phase 2). We conclude that the difference in mean fixation durations depends on whether participants are using a navigation system or not, but not on the different behaviors of the navigation system.

## Discussion

Maps on mobile devices allow navigators to efficiently and effectively find their way across space. Researchers agree that the transformation of assisted navigation from static paper maps to interactive map displays (e.g., navigation systems) that provide information at any time and potentially at any location influences the way we perceive, remember, and interact with our surrounding environment (e.g., Ishikawa et al., 2008; Klippel et al., 2010; Parush et al., 2007). We designed an experiment to study the possible influence of different navigation system designs derived from different levels of automation (Parasuraman et al., 2000) on navigation system use, spatial knowledge acquisition, and gaze behavior during a route-following task. The implemented navigation system behaviors were selected based on research on spatial knowledge acquisition, active learning, and automated systems. Research that emphasizes the importance of engaging a user with the environment (e.g., Gardony et al., 2013) suggests that this active user participation with a navigation system benefits spatial learning during navigation. We developed a new two-phase empirical framework for testing incidental spatial knowledge acquisition in real-world outdoor environments. First, participants were asked to follow a pre-defined route assisted by a navigation system (incidental knowledge acquisition phase). Second, participants were asked to reverse the route without the navigation system (knowledge recall phase). We now discuss our empirical results with regard to the leading research questions and within the context of the research findings reported in the literature. We begin with the behavioral research question:

### How do varying navigation system behaviors (levels of automation) influence (i) navigation performance, (ii) spatial knowledge acquisition, and (iii) gaze behavior during navigation tasks in a real-world outdoor environment?

#### Navigation performance and spatial knowledge acquisition

Research on assisted navigation has studied navigation efficiency (e.g., Lee & Cheng, 2008) or spatial knowledge acquisition (e.g., Gardony et al., 2013; Taylor et al., 2008). According to this research, successful navigators assisted by a navigation system should still make their own decisions, attend to their surroundings, and actively take part in the navigation process because these factors positively affect spatial knowledge acquisition (Chrastil & Warren, 2012; Chung et al., 2016; Kiefer, Giannopoulos, Athanasios Anagnostopoulos, Schöning, & Raubal, 2017; Parush et al., 2007). Based on these studies, we implemented two cognitive processes (i.e., the allocation of attention and self-localization) relevant for wayfinding (Glisky, 2007; Lobben, 2004) with different levels of automation in which either the navigation system or the navigator makes a decision and performs an action (Parasuraman et al., 2000).

The implemented system behaviors with higher levels of human participation aim to increase spatial knowledge acquisition during assisted navigation while still ensuring efficient navigation of their users. Indeed, our results did not reveal any difference in completion time for the assisted route-following phase across the four tested groups. This holds even though two groups had to first decide on and then enter short landmark descriptions into their system three times during Phase 1 and, consequently, needed to stop more often. Still, participants in these groups did not need more time to complete the route-following task compared to participants using systems that selected for them what they should allocate their attention to (e.g., automatic notifications). Thus, navigation system behavior did not influence time to task completion during the assisted part of the experiment. This finding has important implications for developing navigation systems that regulate active user participation (i.e., low level of automation) without harming navigation efficiency.

To determine the impact of system behavior on incidental spatial knowledge acquisition, participants had to reverse the same route without any navigation system assistance, thus using only their spatial knowledge that was incidentally acquired during the assisted route-following phase. We counted a wrong decision at an intersection as a navigation error. During the assisted navigation phase, all participants followed the route without any error. This may not be surprising because they were assisted by a navigation system. During the unassisted phase, the number of errors varied across the four groups.

The different navigation systems implemented the cognitive process “allocation of attention” with two modes at the extreme ends of the spectrum of levels of automation. The two tested modes of the cognitive process “self-localization” exhibit a less pronounced difference of these levels (Fig. 3). We observed a clear difference between the two extreme modes for acquiring spatial knowledge. Both groups with users’ decisions on where to mark landmarks and, thus, where to allocate attention (*AllocHum_LocHum*, allocation of attention and self-localization by human; and *AllocHum_LocSys*, allocation of attention by human and self-localization by system) show 82% success rates in finding the exact same route back. The two groups in which the system allocates users’ attention to landmarks show success rates of 63% (*AllocSys_LocSys*, allocation of attention and self-localization by system) and 38% (*AllocSys_LocHum*, allocation of attention by system and self-localization by human). The fact that so many participants did not find their way back correctly after just 10 min of walking along a simple route may seem surprising. However, these results support the hypothesis of Chrastil and Warren (2012), Parush et al. (2007), and Willis et al. (2009) that activating a user with a location-dependent task (in our case, typing three self-selected keywords into a navigation system) increases spatial knowledge acquisition. In contrast, the two study groups with notification texts (*AllocSys_LocHum* and *AllocSys_LocSys*) who were using a navigation system with a high level of automation show lower success rates. This result seems to confirm findings by Pielot and Rello (2017) and Lee et al. (2014), who demonstrated that system notifications can interrupt an activity. In our case, textual notifications indicated by tactile alarms forced users to focus on their navigation system, rather than the environment, at locations defined by the system. Navigation decisions were taken away from navigators by the system, and thus may have interrupted the process of acquiring spatial knowledge. We further explain this result with the fact that the *AllocSys* groups were forced to switch to the survey perspective at the system’s discretion, while the *AllocHum* groups could maintain the first-person perspective until choosing themselves to make the switch to the survey perspective in order to make a place–action link (as described in Chrastil & Warren, 2012). This explanation aligns with the divided attention literature (Gardony et al., 2013) and the stated cognitive problem of “passive nature of interaction” (Willis et al., 2009). The effects of the two modes of the cognitive process “self-localization” are less pronounced in our study, which may at least in part be explained by the fact that they are similar in their level of automation (levels 8 and 10 in Fig. 1, respectively).

We initially hypothesized that the group faced with the highest level of automation, i.e., in which both decisions on attention allocation and self-localization are made by the device (*AllocSys_LocSys*), would acquire least spatial knowledge. Our results do not really support this hypothesis since the group *AllocSys_LocHum* made the most errors when reversing the route. One possible explanation for this might be that participants in this group interacted with the map more often than the two other groups *AllocHum_LocSys* and *AllocSys_LocSys*. Consequently, the *AllocSys_LocHum* group was frequently switching between the route and survey perspective and seemed to have paid more attention to the navigation system than to the environment compared to the other groups (Ishikawa et al., 2008).

Another explanation may lie in the use of the “GPS on” button to facilitate self-localization. The group *AllocSys_LocHum* used this button significantly more often than the other group with the same option (*AllocHum_LocHum*). The *AllocHum_LocHum* group, which needed to choose and type keywords about landmarks, had this particular task to concentrate on. In contrast, the participants of group *AllocSys_LocHum*, who did not have any other tasks to fulfill, used this button much more than necessary, and consequently had their position displayed on the map for a longer amount of time. Just having the option of pressing this button likely distracted participants in this group more than expected. There are several possible explanations for this result. First, users in the group *AllocSys_LocHum* may indeed have needed repeated confirmations of their current location on the map to successfully find the route during Phase 1. Second, they used the button just because they could or, third, just to offload cognition to the system to reduce “stressful” cognitive activity, which would confirm the findings by Willis et al. (2009). Overall, this result seems consistent with research that found that using smart devices can lead to excessive reliance on the system (Klippel et al., 2010; Parush et al., 2007). Additionally, navigation system use may diminish our navigation skills more generally and, with that, we may not be able to appropriately judge when the use of a navigation interface element becomes optional (Montello, 2009). Third, frequent perspective changes can interrupt the process of allocating enough attention to the surrounding environment.

In general, our results suggest that the use of interactive display elements (e.g., zoom, pan, rotation, GPS button, etc.) invites users to switch between perspectives (Dai et al., 2018) and thus facilitate the division of navigators’ attention between the system and the environment (Gardony et al., 2013). Possibly the groups with a lower level of system automation engaged with the interactive display tools more strategically and in a goal-directed manner. The groups with a higher level of automation did not seem to invest cognitive resources in the navigation task, but rather explored the system’s capabilities and looked for ways to let the system do all the work. Regarding cognitive processes involved in using navigation systems, our findings suggest that differences in the levels of automation of navigation system behavior, specifically, allocating attention and self-localization, affect human navigation behavior and, with it, incidental spatial knowledge acquisition. We further highlight the importance of better understanding the effects of interactive interface components (e.g., display buttons) in navigation system design because they can support, but also hinder, spatial knowledge acquisition, even if they may not affect navigation performance. Our study starts building knowledge to more deeply understand real-world navigation when using navigation systems, as suggested by Dai et al. (2018).

#### Gaze behavior

Because research has found that navigation systems change how humans allocate their attention to the environment and change their landmark selection (Gardony et al., 2013; Ishikawa et al., 2008; Parush et al., 2007; Taylor et al., 2008), we analyzed participants’ gaze behavior during the two experimental phases. Eye movement behavior is one measure of information acquisition (Kiefer et al., 2017) and strategies (Holmqvist et al., 2011). The goal of the eye tracking analysis was to determine the spatio-temporal distribution of participants’ fixation durations along the route.

The results of the fixation duration analysis did not reveal any differences in navigators’ gaze behaviors across navigation system behaviors but, interestingly, did so between the two experimental phases. We found a clear gaze behavior pattern during the assisted route-following task, but no clear patterns emerged during the unassisted route-reversing task. We are unable to systematically identify what participants allocated their attention to during the experiment due to the vast amount of dynamic eye fixation data and the extensively laborious annotation process (Kiefer et al., 2017). However, the applied method generally can tell us something about potential similarity of gaze behaviors across groups in a spatio-temporal context. With our method of segmenting the route according to decision points (i.e., intersections), we were able to detect spatial segments that led to higher mean fixation durations, thus potentially indicating higher cognitive functions, and segments showing lower mean fixation durations, suggesting a potential for increasing visual complexity (Duchowski, 2007). Longer fixation durations in the early segments of the route might indicate that participants are actively becoming familiar with the task, the navigation system, and their surroundings, and, connected to this, higher information processing, or, conversely, that participants had more difficulty to extract information (Goldberg & Kotval, 1999). Longer fixations in this context could also be interpreted as making a clear place–action link, as described in Chrastil and Warren (2012). Interestingly, in segments 6 and 7 of the route, which can be characterized as an unremarkable and quiet street, participants showed the lowest mean fixation durations, with only small variations. According to the literature, spatial scenes with lower fixation durations show decreased cognitive functions and information processing and an increase in visual complexity of the environment. This could mean that unremarkable streets might have led to a switch into a passive navigation mode, i.e., navigators did not pay much attention to the task.

These results suggest that we might be able to relate human behavior to the spatial context during navigation system use, which is a clearly identifiable knowledge gap in the literature (Dai et al., 2018).

With our descriptive summary approach analyzing dynamic mobile eye tracking data, we are able to clearly distinguish different behaviors during different cognitive tasks along a route—this without exactly knowing what features participants attended to. These findings provide further insights into how the allocation of attention might shift between navigation system use and environmental context during navigation.

#### Limitations and future work

We present aggregated results across four navigation system groups. Participants do not show any differences across groups in spatial ability. Consequently, we cannot attribute errors made during Phase 2 to spatial ability. Due to small sample sizes, gender and spatial ability are not further analyzed in this study. Certainly, they could (or should) be assessed and/or controlled for in future navigation studies.

The current studied navigation behaviors rely on visual information only (i.e., map and text). In future work, it would be interesting to include auditory system modalities, as these were found to be beneficial for navigation performance (Klatzky, Marston, Giudice, Golledge, & Loomis, 2006). For example, the system could provide spoken route instructions. Navigation system modes used to reallocate attention could employ auditory modalities to better understand the impact of modality of the presented information. One way of applying these could be that participants are asked to voice-record landmark descriptions, instead of typing them into the system (*AllocHum*), or that the system voices a landmark description when navigators approach their locations, instead of displaying a label on the map (*AllocSys).*

To develop a fuller picture of the gaze behavior, laborious annotations of the eye tracking data are required, which in turn could help us to further verify interpretations of results.

Finally, a similar experiment and data analysis could also be performed in an indoor environment (Riehle, Lichter, & Giudice, 2008) to gain further insights into the influence of varying environmental contexts on navigation behavior.

Overall, our findings have important implications for designing and developing navigation systems that allow for efficient navigation while at the same time supporting acquisition of spatial knowledge. Navigation system design needs to be more thoroughly empirically investigated with respect to levels of automation, modality of information delivery, and where attention is allocated during navigation because these have direct consequences for human navigation behavior and for the ease of acquiring new spatial knowledge.

### Is the experimental framework of an assisted and unassisted navigation phase a valid approach to gather useful data in terms of spatial knowledge acquisition and to allow for a smooth execution of an outdoor experiment?

A second goal of this study was to test a new empirical framework in an outdoor environment, and to use navigation errors at intersections as an indicator of spatial knowledge quality, as Dillemuth (2005), Hund and Gill (2014) and Lovelace and Hegarty (1999) suggested. So far, most research on spatial knowledge acquisition has been carried out in VR setups, under highly controlled conditions (e.g., Brunyé et al., 2014; Gardony et al., 2013). Studies testing spatial knowledge acquisition in the real world, and especially in outdoor environments, are still rare and usually only have small numbers of participants (e.g., Bertel et al., 2017; Frei, Richter, & Fabrikant, 2016). Other wayfinding studies in this domain either tested aspects of usability (Cheverst, Mitchell, & Davies, 2001; Gulliksen et al., 2003; Li & Longley, 2006; Looije, te Brake, & Neerincx, 2007) or of attention allocation (Gardony et al., 2013; Kiefer et al., 2013; Michon & Denis, 2001; Roger, Bonnardel, & Le Bigot, 2009; Ross, May, & Thompson, 2004). Our approach involved a real-world outdoor scenario in which participants’ spatial knowledge was assessed with a route-reversal task that asked participants to find the identical route back. The framework, introduced in Brügger et al. (2016), is similar to the VR study by Karimpur et al. (2016), but was modified for execution in a dynamically changing outdoor urban environment. To be able to apply a real-life scenario (e.g., finding lost keys) in a real-world environment, we let the participants reverse the route and did not use any of the usually applied direction or distance estimation tasks (as, e.g., in Burte & Montello, 2017). What is more, the new proposed framework tests users’ in situ recognition of the environment and allows for efficient experimental execution.

#### Challenges of applying the use-inspired framework in the real world

We conducted the experiments on days with similar weather conditions, and only during daytime. Weather conditions have led to cancellations and rescheduling of trials, which makes outdoor studies time-consuming and more difficult to plan. Because the experimental Phases 1 and 2 were executed within half an hour, the environmental testing conditions can be considered stable, except for moving objects (e.g., cars, pedestrians, etc.). Hence, changes in the environment might have occurred between trials. Another challenge of testing this use-inspired framework is the in situ change of participants’ walking direction between Phase 1 and Phase 2. Because participants experience actual locomotion, are embedded in the real-world environment, and have a novelty of landmark perspectives (Bakdash et al., 2008; Klippel et al., 2010; Montello, 2005; Richter & Winter, 2014), we argue that the change of walking direction is easier to deal with in the real world compared to in virtual environments. Our results confirm that route reversal is a a valid use-inspired task for our purposes as, indeed, two-thirds of the participants were able to reverse the route without any navigation errors.

#### Self-assessed task difficulty of reversing a route

Participants’ perceived task difficulty rating (collected before the navigation task) revealed that they expected the task to be manageable and without many problems. The same task was again rated after they completed the navigation task, and participants found it even easier than expected. This finding is surprising given that more than one-third of the participants made a navigation error. This underestimation of real-world navigation performance is interesting and requires further analysis of subjective perception on navigation performance across system designs.

#### Further development of the experimental framework

Being able to reverse a route one just walked may not represent the only goal of navigation (and neither would pointing back to the origin from a destination), but we contend that the approach we implemented in our study indeed represents an everyday problem. Furthermore, we argue that the framework successfully captures differences in spatial knowledge acquisition without using any of the standard measures, such as pointing or sketch map drawing. Still, to develop a fuller picture of spatial knowledge acquisition during assisted navigation, additional studies in outdoor environments need to further refine our proposed use-inspired framework. For example, it would be useful to develop a classification and quantification scheme for navigation errors (e.g., navigation error and behavior categorization, according to varying spatial contexts), which would allow for more detailed and meaningful analyses of spatial knowledge acquisition and human navigation behavior, beyond this study. Overall, our scenario of finding one’s lost keys on a previously walked route without any navigation assistance does represent a real-world scenario that can be easily applied to different environments and locations, navigation modalities, and other empirical study contexts. Studying spatial knowledge acquisition in real-world outdoor environments makes an important contribution to the challenges of developing “realistic” outdoor studies, beyond the lab-standard of controllability, typically using impoverished environments and lacking realistic contexts. We see this as a benefit, not a limitation.

## Conclusions

Current navigation systems primarily provide information that is useful for navigation performance (efficiency). Due to the way this is implemented in state-of-the-art systems, it typically consumes a navigator’s attention, while in fact navigation systems could be leveraged to better manage attention allocation and self-localization, i.e., could benefit both navigation efficiency and spatial knowledge acquisition. The purpose of this study is to determine how navigation system behavior influences navigation performance, gaze behavior, and incidental spatial knowledge acquisition of pedestrians traversing outdoor environments. We applied a new empirical use-inspired framework that includes a real-world scenario of walking a route assisted by a navigation system and then reversing the same route without the assistance of a navigation system. We have further demonstrated that it is possible to study spatial knowledge acquisition in outdoor environments by recording navigation errors at intersections, using them as one of the indicators of lacking mental spatial representations. Further experimental studies are needed to gain a deeper understanding of the kinds of navigation errors participants make during an unassisted recall phase.

Our approach of deriving navigation system behaviors from levels of automation in cognitive processes relevant for wayfinding is unique, and it extends our knowledge of how navigation system behavior influences human behavior in real-world environments. A greater focus on the combination of cognitive processes during assisted navigation in outdoor environments would enhance our understanding of a navigator’s active role during navigation and possible divided attention effects. The uncovered gaze pattern differences illustrate the opportunities eye tracking data offer to study navigation behavior in real-world and outdoor studies in order to relate human behavior and cognitive activity to spatial context and spatial tasks. We contend that once we find behavior patterns dependent on task, navigation system, and spatial context, we will be better able to design systems that allocate attention based on these patterns in real-time to better handle spatial knowledge acquisition and navigation performance. For example, the system might sense a specific behavior pattern (e.g., intensive, repeated use of a button, or constantly lowering fixation positions) and consequently may force the navigator to keep using his or her own skills by disabling the use of the button or by reminding the user to look up to the environment. With our study, we contributed to the design of future intelligent navigation systems that know where, when, and in which modality cognitive processes should be supported by automation to increase spatial knowledge acquisition during assisted navigation tasks. The task to reverse the same route without a navigation system should then be possible for everybody without any navigation errors.

## Abbreviations

Alloc: Allocation of attention
AOI: Areas of interest
CHF: Swiss francs
CP: Cognitive processes
ETH: Eidgenössische Technische Hochschule
GIS: Geographic information system
GPS: Global positioning system
Hum: Human
Loc: Self-localization
M: Mean
Mdn: Median
SD: Standard deviation
Sys: System
VR: Virtual reality
